# Climate change threatens unique evolutionary diversity in Australian kelp refugia

**DOI:** 10.1038/s41598-023-28301-z

**Published:** 2023-01-23

**Authors:** Matt J. Nimbs, Thomas Wernberg, Tom R. Davis, Curtis Champion, Melinda A. Coleman

**Affiliations:** 1 New South Wales Department of Primary Industries, National Marine Science Centre, Coffs Harbour, NSW Australia; 2 National Marine Science Centre, Southern Cross University, Coffs Harbour, NSW Australia; 3 Oceans Institute and School of Biological Sciences, University of Western Australia, 35 Stirling Highway, Crawley, WA 6009 Australia

**Keywords:** Environmental health, Climate-change ecology, Conservation biology

## Abstract

Climate change has driven contemporary decline and loss of kelp forests globally with an accompanying loss of their ecological and economic values. Kelp populations at equatorward-range edges are particularly vulnerable to climate change as these locations are undergoing warming at or beyond thermal tolerance thresholds. Concerningly, these range-edge populations may contain unique adaptive or evolutionary genetic diversity that is vulnerable to warming. We explore haplotype diversity by generating a Templeton–Crandall–Sing (TCS) network analysis of 119 *Cytochrome C Oxidase* (COI) sequences among four major population groupings for extant and putatively extinct populations only known from herbarium specimens of the dominant Laminarian kelp *Ecklonia radiata* in the south-western Pacific, a region warming at 2–4 times the global average. Six haplotypes occurred across the region with one being widespread across most populations. Three unique haplotypes were found in a deep-water range-edge population off Moreton Island, Queensland, which likely represents both a contemporary and historic refuge during periods of climatic change. Hindcasting *E. radiata* cover estimates using extant data, we reveal that this region likely supported the highest kelp cover in eastern Australia during the last glacial maximum. The equatorward range edge, deep-water kelp populations off Moreton Island represent a genetically diverse evolutionary refuge that is currently threatened by warming and requires prompt ex-situ conservation measures.

## Introduction

Climate change has driven contemporary decline and loss of kelp forests globally (e.g.^1,2^) with an accompanying loss of the considerable ecological and economic values that kelp forests provide^3–5^. Equatorward-range edge kelp populations are particularly vulnerable to climate change as these locations are undergoing warming at or beyond kelp thermal tolerance thresholds^6–8^. Concerningly, these rear edge populations may contain unique adaptive^9,10^ or evolutionary^6^ genetic diversity that is also under threat. Kelp can sometimes persist at lower latitudes, aided by cool water upwelling or in deep-water refugia where they are protected by thermoclines^6,11–14^. Identification of these refuge areas is vital to ensure important genetic diversity is protected and to understand how extant and past climates shape species distribution and evolutionary diversity.

While contemporary genetic diversity can be constrained in low latitude, rear edge populations of temperate species (e.g.^5,8,14,15^), evolutionary diversity may be high due to historical processes^16^. During the Pleistocene glaciation, many marine organisms were only able to persist within ice-free refugia at lower latitudes^17–19^. As temperature and sea level rose and glaciers retreated, species rapidly dispersed poleward and recolonised new areas. The signatures (‘founder effects’) of such distributional changes can be seen in patterns of evolutionary genetic diversity with past low latitude refugia often harbouring high genetic diversity. In contrast, areas that were newly recolonised following extinction or range contractions exhibit lower diversity. Examples of low latitude glacial refugia with high genetic diversity can be found in temperate seaweeds including *Durvillaea antarctica* (Chamisso) Hariot, 1892^20^, *Saccorhiza polyschides* (Lightfoot) Batters, 1902^11^ and *Laminaria ochroleuca* Bachelot de la Pylaie, 1824^21^.

Marine environments in Australia have a stable climatic history and have not directly experienced glaciation events^22^. Rather, Pleistocene cooling caused sea levels to drop and isotherms to shift equatorward around the Australian continent which have also left imprints on species evolutionary diversity^23–25^. For example, a drop in sea level around southern Australia caused the closure of Bass Straight resulting in isolation between the east and southern coastlines and genetic signatures of refugia in many marine species^26–28^. The drop in sea level and temperature associated with glaciation would also have caused vertical migration of species to deeper depths as they followed thermal and light optima. Moreover, widespread extinctions of rocky reef marine organisms were also likely in areas with a narrow continental shelf (e.g. the south eastern Australian coastline) as sea levels fell and the availability of rocky reef dramatically declined (or became non-existent) in many regions^24^.

*Ecklonia radiata* (C. Agardh) J. Agardh, 1848 is the dominant and most widely distributed Laminarian kelp in the Southern Hemisphere^2^ and the only kelp across much of its range, providing the dominant biogenic habitat structure and supporting immense ecological and economic values^3^. Previous research on evolutionary diversity in *E. radiata* in south-eastern Australia^26^ and Western Australia^6^ revealed genetic diversity to be low and dominated by a single cosmopolitan haplotype, which was also found in South Africa and Oman (where it is now extinct)^6^, in addition to a few endemic regional haplotypes. Durrant et al.^26^ suggested that this low diversity in south-eastern Australia was due to rapid colonisation from the northern hemisphere rather than recolonisation following extinction because no refugial areas with higher genetic diversity were found. However, latitudes lower than 35° S on Australia’s east coast have the densest contemporary *E. radiata* forests^29^ and represents the area where Pleistocene temperature would have been optimal for climate refugia to occur^30^ but have never been sampled.

The low latitudinal limit of distribution of *E. radiata* on Australia’s east coast is at Moreton Island (27° S, Queensland) where this species only occurs on cooler, deep water reefs (30-80 m) below thermoclines^14,31^. This region is also interesting from an historical climate change perspective. Pleistocene glaciation caused large drops in sea level and temperature that would likely have caused significant shifts in both the latitudinal and vertical distribution, including colonisation of what are currently deeper rocky reefs by *E. radiata*. At this time the Moreton Island region would have experienced optimal temperatures (21–24 °C) to support *E. radiata* populations^30^. Moreover, widespread local extinctions were also likely as the already narrow continental shelf (and availability of rocky reef) became markedly reduced south of Moreton Island with a fall in sea level^24,32,33^. As such, the extant deep water Moreton Island populations may represent both an historical and contemporary refuge from changing climates and harbour high genetic diversity. *Ecklonia radiata* also historically occurred on the isolated Lord Howe Island (31° S) with two herbarium specimens confirming its existence there. Further north, a remote deep-water population of *E. radiata* is present east of Norfolk Island (29° S)^34^ where a single herbarium sample exists (Auckland Museum—AK146440) from 1930, and a recent specimen was dredged up during fishing (Mark Scott, pers. comm). There have been no contemporary confirmations of extant populations on Lord Howe Island despite widespread habitat surveys. Consequently, we presume that this population is extinct. This low latitude island may also represent a lost refugia that once harboured unique or high genetic diversity.

Given that eastern Australia is warming at a rate that is approximately four times greater than the global average^35–37^ which has driven contemporary declines in *E. radiata*^7^, it is pertinent to identify evolutionary refugia to better understand how climate drives the distribution of this critical species and to protect unique genetic diversity. While current understanding of *E. radiata* population genetics indicates that these low latitude populations have low neutral genetic diversity due to limited extant connectivity^15,38^ but have been selected for higher thermal tolerance^10^, it remains unknown how historical processes have shaped this foundation species. We use the mitochondrial *Cytochrome C Oxidase* (COI) marker to examine evolutionary genetic (haplotype) diversity among extant and putatively extinct *E. radiata* populations from across its full latitudinal range in eastern Australia and across the south-west Pacific. We predicted that haplotype diversity would be higher at lower latitudes due to their potential role as historical refugia.

## Materials and methods

### Ecklonia radiata specimens

**Figure 1:**
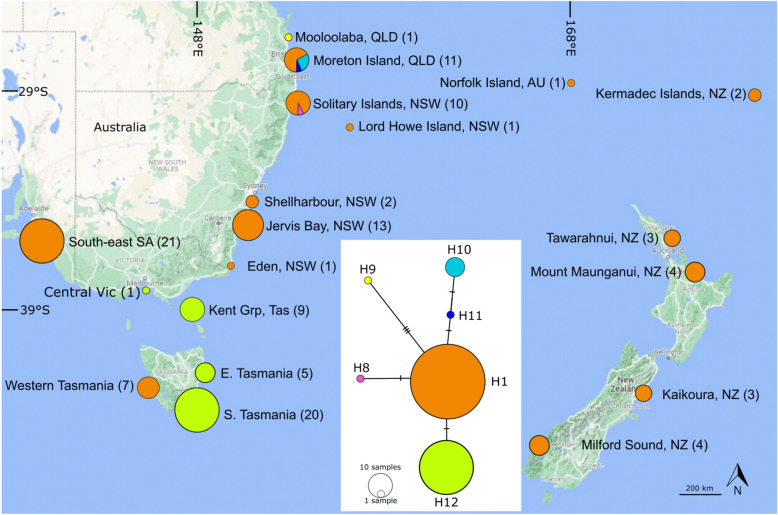
Haplotype network and haplotype distribution of *Ecklonia radiata* based on COI barcode markers from the south-western Pacific. Colours represent different haplotypes; circle size is representative of sample size (sample size also provided in parentheses after sampling location name) and network link hatch marks represent base pair substitutions. Haplotype numbers correspond to Coleman et al.^6^. Map created in Inkscape v1.2 (https://inkscape.org/release/inkscape-1.2/).

To examine patterns of evolutionary diversity of *Ecklonia radiata* across the south-west Pacific, we use a mix of freshly collected *E. radiata* (collected as in Ref.^15^) and accessed herbarium specimens of rare (Norfolk Island; only one sample in herbaria globally) and the putatively extinct population at Lord Howe Island (only two samples in herbaria globally), (Supplementary Information, see Fig. 1 for locations). First, we obtained subsamples of dried herbarium specimens of *E. radiata* from Norfolk Island collected in 1930 (Auckland Museum, New Zealand (NZ), accession AK146440), and Lord Howe Island, collected in June 1933 (National Herbarium of New South Wales (NSW), Sydney, accession NSW817202). We also obtained a Lord Howe Island sample collected in 1966 (Melbourne Herbarium MEL16690 but it did not produce DNA that amplified. We also accessed thallus and stipe material from a single deep-water specimen, collected from 70 m off Moreton Island, QLD in August 2017 (this is the only sample available in herbaria) that was held at the QLD herbarium (AQ1019163) fixed in 10% formalin, and two deep water, dried herbarium specimens from the Kermadec Islands (Auckland Museum, NZ).

Fresh samples of *Ecklonia radiata* were collected from several locations in south-eastern Australia (Moreton Island, Solitary Islands, Shellharbour, Eden) and New Zealand (Tawarahnui Peninsula, Mount Maunganui, Kaikoura and Milford Sound) (see Supplementary Information for location details). Samples were collected by hand from rocky reefs by diving or snorkelling and either snap frozen in liquid nitrogen (as in Ref.^10^) or dried in silica gel (as in Ref.^15^). One specimen collected at Mooloolaba, Queensland, 60 km north of the known extant range of *E. radiata*, was collected as dried beach wrack. As the sampling methods for herbarium specimens are largely unknown, we treat their geographic origin as that originally stated by the collector. All methods were performed in accordance with relevant guidelines and regulations; samples were collected under scientific permits issued by state or federal governments permits (P01/0059(A)-2.0 and QS2018/MAN471); and herbarium specimens were obtained under agreements with each herbarium/institution.

### DNA extraction and gene amplification

For fresh, dried and preserved herbarium specimens approximately 50 mg of tissue was used for extraction. For the single formalin fixed sample, 150 mg of internal stipe tissue was used for extraction as thallus tissue failed to produce DNA or amplify. DNA was isolated using (a) a combined CTAB and SDS protocol^39^ or (b) the Qiagen DNeasy Plant Pro kit, following the manufacturer’s instructions. For both protocols the tissue lysis step was extended to 10 days incubation at 65 °C with samples vortexed twice daily. Extracted DNA from fresh material was purified using a Qiagen DNeasy PowerClean Pro clean-up kit following the manufacturer’s protocol. Purified DNA that was deemed of sufficient quality and quantity (at least ~ 3 ng/µL with > 600 bp molecular weight) was PCR amplified for a portion of the mitochondrial cytochrome* c* oxidase 1–5′ barcoding region (COI) with the previously published primers, Gaz F2 (5′ CCAACCAYAAAGATATWGGTAC 3′) and Gaz R2 (5′ GGATGACCAAARAACCAAAA 3′)^40^ in 50 µL volumes using the Invitrogen Platinum II Hot-start PCR Master-Mix (2x). To reduce the risk of contamination, DNA extractions and PCRs were done in separate areas with their own pipettes and equipment, and PCR set-up was carried out using freshly opened packages of consumables with all work carried out in a UV-sterilised laminar flow cabinet^41,42^. A PCR cocktail was prepared as per the manufacturer’s instructions (7.5 µL template) and cycled in an Eppendorf Mastercycler Nexus Gradient thermocycler. Visualisation of PCR products was carried out using a 2% agarose plate to determine the reaction success for each marker. Many samples failed to amplify using the Invitrogen Platinum II Hot-start kit, so we also amplified 1.0 µL of DNA extract in a repeat PCR in a Thermo Scientific Phire Plant Direct PCR Master Mix kit following the manufacturer’s ‘dilute and store’ protocol. This kit is designed to amplify DNA directly from plant tissues without the need for extraction and purification. The polymerase is optimised to tolerate PCR inhibitors such as polyphenols and polysaccharides, and requires very small amounts of DNA template. We have found it to work well for poorer-quality, low-concentration, herbarium derived DNA. In accordance with the Thermo Scientific Phire Plant Direct PCR Master Mix kit manufacturer instructions, a gradient series PCR was performed, using purified DNA, to determine the optimal annealing temperature for the primer/template pair (53.5 °C). PCR cycling consisted of an initial denaturation for 5 min at 98 °C, followed by 45 cycles of: 5 s at 98 °C, 5 s at 53.5 °C and 45 s at 72 °C. A final extension was carried out for 1 min at 72 °C. Successful amplicons that produced a clear band on a gel with a clear negative control were outsourced for purification and sequencing at the Australian Genomic Research Facility (AGRF), Sydney. We note that for the herbarium samples it often took two to three repeat extractions and/or three to four PCR reactions tweaking master mixes, template volumes and thermocycler conditions to obtain suitable product for sequencing.

Forward and reverse sequence reads were de novo assembled using Geneious Prime 11.1.5^41,42^ and edited by eye. A further 76 COI sequences derived from *E. radiata* from the south-western Pacific^26^ were retrieved from GenBank (Supplementary Information) and were aligned with the newly-generated sequences using the MUSCLE algorithm included in Geneious Prime using default settings^43^. Forward and reverse primers were trimmed from sequences and two data quality checks were carried out: a BLAST search^44^ to compare sequences with those stored in NCBI-accessible databases to verify that correct genetic markers had been amplified, and protein translation on the complete alignment based on the standard genetic code.

### Haplotype diversity and network

The COI nucleotide alignment of *Ecklonia radiata* sequences was exported from Geneious Prime in NEXUS format for use in haplotype diversity analysis and reconstruction of an haplotype network. Diversity was analysed using the DNA Sequence Polymorphism package (DNAsp)^45^ where samples were aggregated into regional populations for visualisation and analysis based on shared haplotype identity: South Australia, Victoria and Tasmania (SA, Vic Tas); New South Wales (NSW); Queensland (QLD); and Lord Howe Island, Norfolk Island, Kermadec Islands and New Zealand (LHI, NI, KI, NZ). A Templeton-Crandall-Sing (TCS) haplotype network^46^ was reconstructed using PopART software^47^. The resultant network graphic was improved and overlaid on a vector map using InkScape 1.1^48^ Remote or deep-water locations made sample collection difficult, and for this reason sample numbers for some locations are low. As a result, conclusions based on sampling from these locations need to be regarded with caution because there may be unsampled haplotypes.

### Last glacial maxima data

To illustrate the likely temperature-driven latitudinal shift in kelp distributional patterns between the LGM and present, we used current day estimates of kelp cover^29^ given established links between population size, genetic diversity, and the a priori assumption that regions with high kelp cover would likely have supported high genetic diversity^38,49,50^. Here, the relationship between kelp cover and mean annual sea surface temperature throughout eastern Australia (18–38° S) was derived from field surveys undertaken over a ~ 1000 km latitudinal gradient and remotely-sensed ocean surface temperature (see Davis et al.^29^). This relationship was used to predict and compare the distributions of mean kelp cover estimated for the present-day and the LGM. Present-day cover was estimated using a 25-year average of mean annual sea surface temperature encompassing the period 1996–2020 sourced from the Copernicus Marine Environment Monitoring Service (https://marine.copernicus.eu; product #010_011). Kelp cover at the LGM was estimated using mean annual sea surface temperature data reconstructed for the oceans around Australia using planktonic foraminifera assemblages by Barrows and Juggins^30^. Estimated kelp cover for the present-day and LGM were then averaged latitudinally within the maximum range of depths (i.e. 0–80 m^2,14^) that *E. radiata* is known to occur. Given that sea level was approximately 120 m lower at the LGM, the analysis for this period encompassed continental shelf that is 120–200 m deep at the present-day sea level (representing 0–80 m depth at the LGM). However, this analysis does not take into account any cross-shelf variability in habitat availability (rocky reef) that may have occurred as sea level fell, due to uncertainty regarding local geomorphological processes and the subsequent distribution of rocky substrate during the LGM. We also acknowledge that factors other than ocean temperature (e.g. nutrient and light availability and biological interactions) contribute to the realised distribution of kelp cover^29^ and these were not incorporated here.

## Results

A total of 43 COI sequences (658 bp) were newly-generated for this study, representing the first COI barcode dataset for *E. radiata* from northern NSW, southern Queensland, Lord Howe Island, Norfolk Island and New Zealand (Supplementary Information). MegaBLAST searches of newly-generated COI sequences (including the historic herbarium samples) confirmed that all samples were *E. radiata* (> 98%).

### Diversity and haplotype networks

A total of six haplotypes were present among *E. radiata* samples from the study area (H1 and H8 to12) (Note: we follow Coleman et al.^6^ in the allocation of haplotype numbers to maintain clarity among *Ecklonia radiata* haplotypes, wherein H1 is widely distributed across the Southern Hemisphere and H8–12, nominated here, are private to the south-west Pacific. H 2–7 are *E. radiata* haplotypes that are not present here but in Southern Africa and Oman^6^). Although nucleotide differences among haplotypes were small (Table 1), the most diverse population was in southern Queensland (Hd = 0.636) followed by Victoria, Tasmania and South Australia (Hd = 0.486) and NSW (Hd = 0.077). A single, widely distributed haplotype H1 (*n* = 74), was found across the entire study area from south-eastern South Australia, south to western Tasmania, north along the NSW coast into southern QLD east to Norfolk Island (NI), the Kermadec Islands (KI), south to the north island of New Zealand (NZ) and at Lord Howe Island (LHI) (Fig. 1).

**Table 1:** Diversity metrics for *Ecklonia radiata* in the south-western Pacific.

Population	N	K	S	π	H	Hd
SA, Vic, Tas	63	0.486	1	0.00087	2	0.486
NSW	26	0.077	1	0.00014	2	0.077
QLD	12	1.515	5	0.00272	4	0.636
LHI, NI, KI, NZ	18	0.0000	0	0.00000	1	0.000
All	119	0.744	7	0.00133	6	0.543

*N* number of sequences in population set, *K* average number of nucleotide differences, *S* number of segregating sites, *π* nucleotide diversity, *H* number of haplotypes, *Hd* haplotype diversity.

One haplotype was restricted to cool-temperate waters: H12 (*n* = 35) found in Bass Strait and south-eastern Tasmania (labelled as H1 haplotype in Durrant et al.^26^). Conversely, four haplotypes were restricted to subtropical waters north of 30°S. One haplotype (H8 (*n* = 1)) was found in northern NSW (Solitary Islands), two were from Moreton Island, QLD (H10 (*n* = 7) and H11 (*n* = 1)) and a single haplotype that was three base-pair substitutions different from H1, H9 (*n* = 1), was found at Mooloolaba, QLD (Fig. 1).

### Predicting cover of Ecklonia at the LGM

**Figure 2:**
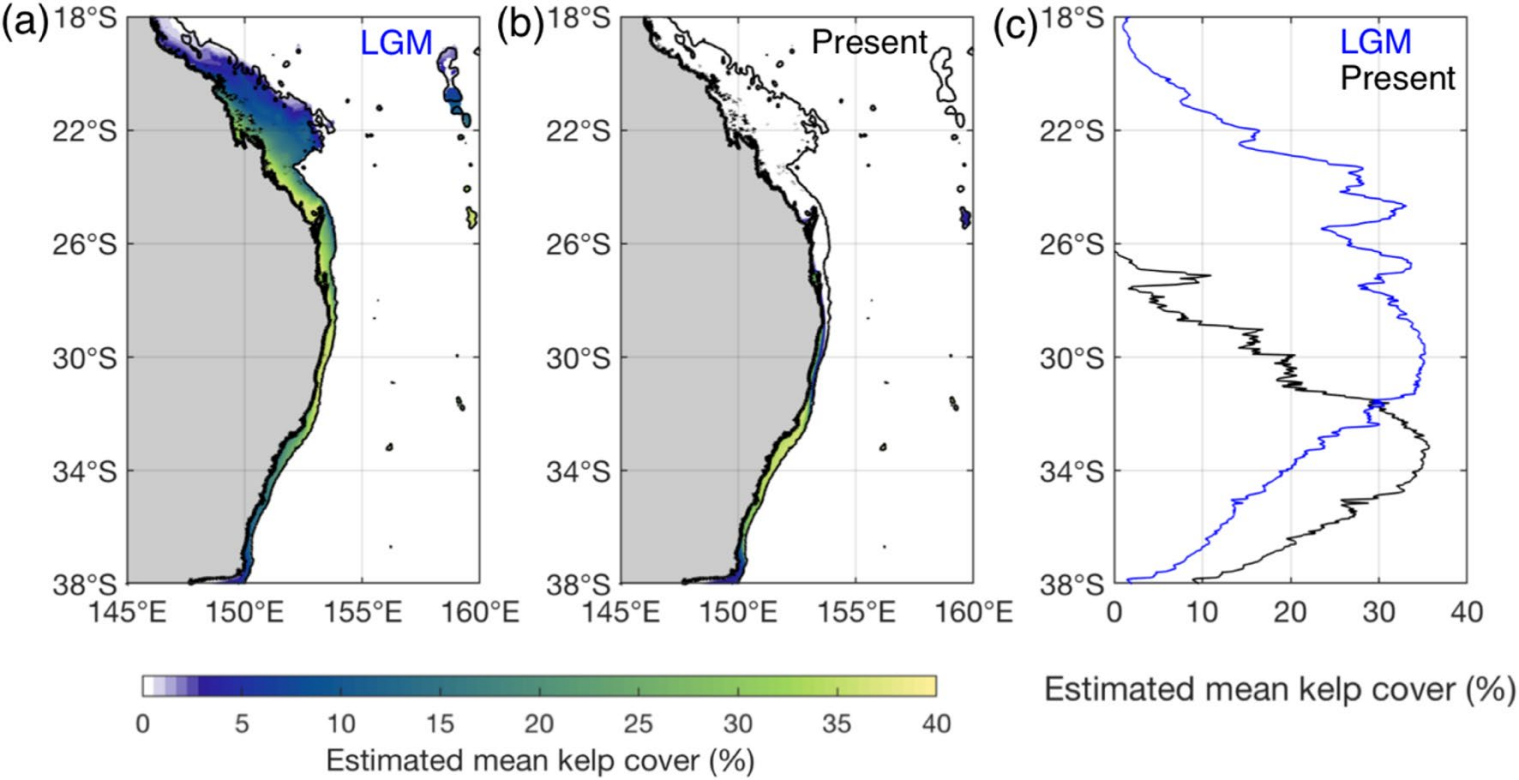
Estimated mean cover (%) of *Ecklonia radiata* predicted for the (**a**) last glacial maximum (LGM) based on reconstructed mean annual sea surface temperature data from Barrows and Juggins^30^, and (**b**) present-day based on a 25-year average of mean annual sea surface temperature encompassing the period 1996–2020. Panel (**c**) displays the latitudinal average of estimated mean *E. radiata* cover at the LGM (blue data) and present-day (black data) within the maximum range of depths (0–80 m) that kelp occurs. Spatial predictions of mean *E. radiata* cover presented in panels (**a,b**) extend across the full continental shelf (i.e. 0–200 m depth range) to aid visual interpretation of the latitudinal trends presented, but data shown in panel (**c**) reflect the estimated mean kelp cover within the maximum range of depths (0–80 m) that kelp occurs. Relative to present-day sea level, the present-day analysis (black data) encompasses the 0–80 m portion of the continental shelf, while the LGM analysis (blue data) encompasses the 120–200 m portion of the continental shelf (i.e. 0–80 m at LGM sea level).

Modelling revealed that during the last glacial maximum the optimal mean annual temperature isotherm for *E. radiat*a [~ 23 °C]^29^ may have been located between Rockhampton (~ 21° S) and Moreton Island (~ 27°S)^30,51^ and, consistent with contemporary biomass distributions^29^_,_ this region would likely have supported dense populations of *E. radiata* (Fig. 2). High average kelp cover would have extended between 31 and 24° S and have been similar to extant regions around 34° S, a, equatorward shift of 3 to 10 degrees of latitude.

## Discussion

Warm, rear edge populations of temperate species can often harbour high and unique evolutionary genetic diversity as they represent historic refugia from past climates^11,20,21^. We examined whether such deep-water rear-edge kelp populations off eastern Australia may have been refugia during periods of glaciation when sea levels fell, temperature cooled and the availability of rocky reef declined along this coast^23–25^. Indeed, we found very high and unique evolutionary diversity in these deep, rear-edge populations of *E. radiata* compared to anywhere else sampled throughout the distribution of this species^6^. This diversity resided primarily in a small, isolated population that is restricted to deep water (> 30 m) off Moreton Island corroborating the likely role of this reef as a refuge under past climates. Moreover, unique diversity was found in a single specimen of beach wrack ~ 60 km north of the known extant range of *E. radiata* in Mooloolaba, suggesting that undiscovered deep-water populations may exist along this coast. This is supported by spatial modelling based on ecophysiological and oceanographic data that indicated that during the LGM, kelp forests were likely to have been extensive and dense in south-eastern Queensland.

### Historical refuge and distribution of *Ecklonia radiata*

The relatively high and unique kelp genetic diversity off south-eastern Queensland (Moreton Island and Mooloolaba) suggests that these rocky reefs were likely an historic refuge for temperate marine organisms under past climates when waters were cooler and sea levels lower. During the last glacial maximum (LGM; 21,000 ± 2000 cal yr BP) the optimal mean annual temperature isotherm for *E. radiat*a [~ 23 °C]^29^ was estimated to be located between Rockhampton (~ 21° S) and Moreton Island (~ 27°S)^30,52^ and, consistent with contemporary biomass distributions^29^_,_ this region would likely have supported large and dense populations of *E. radiata* (Fig. 2). At this time, sea levels were approximately 65 to 125 m lower than present mean sea level^25^ and within 60 km of the present coastline^23^ limiting *E. radiata* to the few rocky reefs that exist on the shelf (e.g. China Wall, Henderson’s Reef) and areas with unconsolidated rocky substrates^31^. Further, most of the rocky reef south of Moreton Island where the continental shelf is very narrow (only ~ 25 km wide)^53^, would have emersed, limiting habitat availability and restricting the distributions of rocky reef marine organisms along much of the NSW coast.

Indeed, the dominance of haplotype H1 throughout much of the present-day range of *E. radiata* suggests rapid recolonisation from refugia following glacial extinction or range contraction. Post-glacial warming would likely have permitted rapid poleward dispersal and expansion including across the Tasman Sea past Lord Howe and Norfolk Islands to New Zealand into the present-day latitudinal range for *E. radiata* (some 10° of latitude south of LGM isotherms). The concomitant rise in sea level of approximately 60–120 m flooded large areas of what is now shallow continental shelf waters off NSW where the newly available rocky benthos provided space for the primary succession of this pioneer genotype. As discussed by Hewitt^54^ pioneer genotypes (H1 haplotype) would dominate this new range and any subsequent genotypic dispersal is less likely to establish due to maximal carrying capacity and competition (i.e. high-density blocking) within the established populations^52,55^.

As glaciers further retreated, and marine isotherms shifted further south facilitating present day species distributions, the refugial *E. radiata* populations in southern Queensland would have become increasingly thermally stressed over timescales of weeks to months as temperatures exceeded the thermal threshold for *E. radiata* (~ 26 °C^2^) except at depth. Indeed, observations suggest that *E. radiata* occasionally recruit into shallower areas around Moreton Island in cooler winter months (~ 20 °C) but rapidly succumb to warmer temperatures in spring and summer when temperatures exceed ~ 27 °C (pers. obs. T. Stevens). Similarly, Lord Howe (~ 31° S, SST 19–26 °C), where *E. radiata* is putatively extinct, sits within the flow of the warm east Australian current where temperatures now mostly exceed thermal tolerances in summer^56^. Norfolk Island (~ 29° S) is slightly cooler (SST 18–25 °C) which allows *E. radiata* to exist in isolated deep-water habitats. Surveys for *E. radiata* in deep water refugia are warranted on Lord Howe Island (to assess presence/absence) and Norfolk Island (to map geographic extent), in order to further elucidate how past and present climate shapes the distribution of this key kelp and to prioritise areas for conservation.

On a broader scale, *Ecklonia radiata* populations in the Indian Ocean exhibit similar haplotype diversity (Hd = 0.586, 7 haplotypes) to the south-Western Pacific (Hd = 0.526, 6 haplotypes). The H1 haplotype extends westward into the Indian Ocean from southern Western Australia, west to southern Africa, and, historically at Oman in the north^6^. In several range edge populations at the Cape Province of South Africa and at the Abrolhos Islands in Western Australia, the widespread H1 is absent, replaced by regionally-unique, private haplotypes^6^ suggesting these areas have been isolated historically or physically (Abrolhos, Western Australia). These patterns in evolutionary diversity contrast with contemporary patterns in neutral diversity in this species^15,38^ and other macroalgal taxa^9,57^ which decline with increasing latitude along the same coastline. This is due to the strong poleward flow of the East Australian Current that isolates temperate macroalgal populations at low latitudes. However, low diversity at low latitudes can also be adaptive^9,10^ and these populations have loci under selection linked to higher and more variable temperatures, including those off Moreton Island (Minne et al. unpbl. data). Understanding the relationships between evolutionary and contemporary patterns of diversity would benefit from genotyping samples using both COI and SNP markers as well as thermal stress experiments on selected haplotypes.

While utilising herbarium collections to examine patterns of genetic diversity is beginning to gain traction for terrestrial plants^58^), this study is one of the few to successfully amplify and sequence DNA from old algal herbarium samples. Coleman et al.^6^ successfully amplified DNA from 47 year old samples from Oman stored in three different herbaria. Similarly, Goff and Moon^59^ amplified DNA from 29 year old samples and Nahor et al.^60^ from a 117 year old sample. We also successfully extracted DNA from a formalin preserved sample. We note that this remains a challenging task. Successful DNA extraction and PCR amplification often required multiple attempts, tweaking both extraction and PCR protocols. We recommend the Themo Fisher Phire Plant Direct PCR kit which is designed to amplify small amounts of DNA in the presence of plant-derived PCR inhibitors. Moreover, extracting DNA from formalin preserved specimens may be easier using the internal portions of the thicker thallus or stipe in which formalin does not penetrate as deeply. Herbariums are an underutilised resource for examining historical genomic patterns yet hold great promise for uncovering the drivers of seaweed distribution and climate impacts^61^.

Contemporary climate change is threatening the high and unique genetic diversity found among eastern Australian low-latitude range-edge populations with warming causing declines of *E. radiata* along this coastline^7^. Moreover, projections suggest further declines and range contractions of kelp under future climate scenarios^29^ as has occurred in range edge *E. radiata* populations globally^6,8^. While it is unlikely that in situ protection could halt declines of rear edge kelp populations under scenarios of warming, their unique genetic diversity could be protected and studied ex situ in culture banks for use in restoration, hybridisation^62^ or assisted adaptation strategies.

## Supplementary Information

Supplementary Information.

## Data Availability

The datasets generated and/or analysed during the current study are available in the NCBI GenBank repository at https://www.ncbi.nlm.nih.gov/nuccore. GenBank accession numbers for each sequence are available in Supplementary Information.
