# Supplementary material for: Climate change threatens unique evolutionary diversity in Australian kelp refugia
Source: Sci Rep. 2023 Jan 23;13:1248. doi: 10.1038/s41598-023-28301-z (PMC9870953; doi:10.1038/s41598-023-28301-z)
Supplement: Supplementary file 1 — Supplementary Information. [file 41598_2023_28301_MOESM1_ESM.doc]

**Supplementary Information.** Details of *Ecklonia* *radiata* samples analysed in this study and COI sequences retrieved from GenBank.

| **Species** | **Location (#sequences)** | **Isolate** | **Depth** | **Lat/Long** | **Collected by** | **Year** | **Population** | **GB Accession** | **Source** |
| --- | --- | --- | --- | --- | --- | --- | --- | --- | --- |
| *Ecklonia radiata* | Kermadec Islands, NZ | AK366714 | ≥70m | 34.0° S, 122.8° E | R.V.Tangaroa | 2016 | LHI, NI, KI & NZ | OM643807 | This paper |
| *Ecklonia radiata* | Kermadec Islands, NZ | AK366710 | ≥70m | 34.0° S, 122.8° E | R.V.Tangaroa | 2016 | LHI, NI, KI & NZ | OM643808 | This paper |
| *Ecklonia radiata* | Tāwarahnui, NZ | NZ1 | Intertidal | 36.3° S, 174.7° E | M. Nimbs | 2019 | LHI, NI, KI & NZ | OM643826 | This paper |
| *Ecklonia radiata* | Tāwarahnui, NZ | NZ2 | Intertidal | 36.3° S, 174.7° E | M. Nimbs | 2019 | LHI, NI, KI & NZ | OM643827 | This paper |
| *Ecklonia radiata* | Tāwarahnui, NZ | NZ7 | Intertidal | 36.3° S, 174.7° E | M. Nimbs | 2019 | LHI, NI, KI & NZ | ON815352 | This paper |
| *Ecklonia radiata* | Mt Maunganui, NZ | MtM10 | 2m | 37.6° S, 176.2° E | R. Lawton | 2021 | LHI, NI, KI & NZ | OM643810 | This paper |
| *Ecklonia radiata* | Mt Maunganui, NZ | MtM9 | 2m | 37.6° S, 176.2° E | R. Lawton | 2021 | LHI, NI, KI & NZ | OM643809 | This paper |
| *Ecklonia radiata* | Mt Maunganui, NZ | MtM11 | 2m | 37.6° S, 176.2° E | R. Lawton | 2021 | LHI, NI, KI & NZ | ON815351 | This paper |
| *Ecklonia radiata* | Mt Maunganui, NZ | MtM2 | 2m | 37.6° S, 176.2° E | R. Lawton | 2021 | LHI, NI, KI & NZ | ON815350 | This paper |
| *Ecklonia radiata* | Kaikoura, NZ | NZK1 | 2m | 42.3° S, 173.6° E | S. & P. Hart | 2021 | LHI, NI, KI & NZ | ON815343 | This paper |
| *Ecklonia radiata* | Kaikoura, NZ | NZK2 | 2m | 42.3° S, 173.6° E | S. & P. Hart | 2021 | LHI, NI, KI & NZ | ON815344 | This paper |
| *Ecklonia radiata* | Kaikoura, NZ | NZK3 | 2m | 42.3° S, 173.6° E | S. & P. Hart | 2021 | LHI, NI, KI & NZ | ON815345 | This paper |
| *Ecklonia radiata* | Milford Sound, NZ | NZMIL1 | 5+m | 44.6° S, 167.8° E | S. & P. Hart | 2021 | LHI, NI, KI & NZ | ON815346 | This paper |
| *Ecklonia radiata* | Milford Sound, NZ | NZMIL2 | 5+m | 44.6° S, 167.8° E | S. & P. Hart | 2021 | LHI, NI, KI & NZ | ON815347 | This paper |
| *Ecklonia radiata* | Milford Sound, NZ | NZMIL3 | 5+m | 44.6° S, 167.8° E | S. & P. Hart | 2021 | LHI, NI, KI & NZ | ON815348 | This paper |
| *Ecklonia radiata* | Milford Sound, NZ | NZMIL4 | 5+m | 44.6° S, 167.8° E | S. & P. Hart | 2021 | LHI, NI, KI & NZ | ON815349 | This paper |
| *Ecklonia radiata* | Norfolk Island, AU | AK146440 | 38m | 29.0° S, 167.9° E | A. Foster | 1930 | LHI, NI, KI & NZ | OM643814 | This paper |
| *Ecklonia radiata* | Lord Howe Island, AU | NSW817202 |  | 31.5° S, 159.0° E | L. Dunbar | 1933 | LHI, NI, KI & NZ | OM643811 | This paper |
| *Ecklonia radiata* | Moreton Island, QLD, AU | CW6 | 30m | 27.0° S, 153.4° E | M. Coleman | 2018 | QLD | OM643831 | This paper |
| *Ecklonia radiata* | Moreton Island, QLD, AU | CW8 | 30m | 27.0° S, 153.4° E | M. Coleman | 2018 | QLD | OM643832 | This paper |
| *Ecklonia radiata* | Moreton Island, QLD, AU | CW11 | 30m | 27.0° S, 153.4° E | M. Coleman | 2018 | QLD | OM643833 | This paper |
| *Ecklonia radiata* | Moreton Island, QLD, AU | CW16 | 30m | 27.0° S, 153.4° E | M. Coleman | 2018 | QLD | OM643837 | This paper |
| *Ecklonia radiata* | Moreton Island, QLD, AU | CW17 | 30m | 27.0° S, 153.4° E | M. Coleman | 2018 | QLD | OM643834 | This paper |
| *Ecklonia radiata* | Moreton Island, QLD, AU | CW24 | 30m | 27.0° S, 153.4° E | M. Coleman | 2018 | QLD | OM643835 | This paper |
| *Ecklonia radiata* | Moreton Island, QLD, AU | CW25 | 30m | 27.0° S, 153.4° E | M. Coleman | 2018 | QLD | OM643812 | This paper |
| *Ecklonia radiata* | Moreton Island, QLD, AU | CW28 | 30m | 27.0° S, 153.4° E | M. Coleman | 2018 | QLD | OM643830 | This paper |
| *Ecklonia radiata* | Moreton Island, QLD, AU | CW29 | 30m | 27.0° S, 153.4° E | M. Coleman | 2018 | QLD | OM643813 | This paper |
| *Ecklonia radiata* | Moreton Island, QLD, AU | CW30 | 30m | 27.0° S, 153.4° E | M. Coleman | 2018 | QLD | OM643836 | This paper |
| *Ecklonia radiata* | Moreton Island, QLD, AU | AQ1019163 | +80m | 27.9° S, 153.9° E | T. Stevens | 2017 | QLD | OM643806 | This paper |
| *Ecklonia radiata* | Mooloolaba, QLD, AU | Mool1 | Wrack | 26.6° S, 153.1° E | M. Coleman | 2018 | QLD | OM643805 | This paper |
| *Ecklonia radiata* | Solitary Is., NSW, AU | SS1 | 15m | 30.2° S, 153.2° E | M. Coleman | 2018 | NSW | OM643817 | This paper |
| *Ecklonia radiata* | Solitary Is., NSW, AU | SS2 | 15m | 30.2° S, 153.2° E | M. Coleman | 2018 | NSW | OM643818 | This paper |
| *Ecklonia radiata* | Solitary Is., NSW, AU | SS5 | 15m | 30.2° S, 153.2° E | M. Coleman | 2018 | NSW | OM643819 | This paper |
| *Ecklonia radiata* | Solitary Is., NSW, AU | SS7 | 15m | 30.2° S, 153.2° E | M. Coleman | 2018 | NSW | OM643820 | This paper |
| *Ecklonia radiata* | Solitary Is., NSW, AU | SS10 | 15m | 30.2° S, 153.2° E | M. Coleman | 2018 | NSW | OM643821 | This paper |
| *Ecklonia radiata* | Solitary Is., NSW, AU | SS18 | 15m | 30.2° S, 153.2° E | M. Coleman | 2018 | NSW | OM643822 | This paper |
| *Ecklonia radiata* | Solitary Is., NSW, AU | SS20 | 15m | 30.2° S, 153.2° E | M. Coleman | 2018 | NSW | OM643823 | This paper |
| *Ecklonia radiata* | Solitary Is., NSW, AU | SS26 | 15m | 30.2° S, 153.2° E | M. Coleman | 2018 | NSW | OM643824 | This paper |
| *Ecklonia radiata* | Solitary Is., NSW, AU | SS29 | 15m | 30.2° S, 153.2° E | M. Coleman | 2018 | NSW | OM643829 | This paper |
| *Ecklonia radiata* | Solitary Is., NSW, AU | SS30 | 15m | 30.2° S, 153.2° E | M. Coleman | 2018 | NSW | OM643825 | This paper |
| *Ecklonia radiata* | Shellharbour, NSW, AU | SH1 | 3m | 34.5° S, 150.8° E | M. Coleman | 2018 | NSW | OM643815 | This paper |
| *Ecklonia radiata* | Shellharbour, NSW, AU | SH2 | 3m | 34.5° S, 150.8° E | M. Coleman | 2018 | NSW | OM643816 | This paper |
| *Ecklonia radiata* | Jervis Bay, NSW, AU (5) | Drumsticks01-05 |  | 35.0° S, 150.8° E |  |  | NSW | KT15799-803 | (Durrant et al., 2015) |
| *Ecklonia radiata* | Jervis Bay, NSW, AU (4) | CallalaReef01-04 |  | 35.0° S, 150.7° E |  |  | NSW | KT158795-8 | (Durrant et al., 2015) |
| *Ecklonia radiata* | Jervis Bay, NSW, AU (4) | OuterTubes01-04 |  | 35.0° S, 150.8° E |  |  | NSW | KT158851-4 | (Durrant et al., 2015) |
| *Ecklonia radiata* | Eden, NSW, AU | E32 | 5m | 37.0° S, 149.9° E | M. Coleman | 2018 | NSW | OM643828 | This paper |
| *Ecklonia radiata* | Kent Group, Tas. AU (4) | ErithIsland01-04 |  | 39.4° S, 147.3° E |  |  | SA, Vic & TAS | KT158808-11 | (Durrant et al., 2015) |
| *Ecklonia radiata* | Kent Group, Tas., AU (5) | SquallyCove01-05 |  | 39.5° S, 147.3° E |  |  | SA, Vic & TAS | KT158857-61 | (Durrant et al., 2015) |
| *Ecklonia radiata* | Eastern Tasmania, AU (3) | Oakhampton01-03 |  | 42.5° S, 147.9° E |  |  | SA, Vic & TAS | KT158848-50 | (Durrant et al., 2015) |
| *Ecklonia radiata* | Eastern Tasmania, AU (2) | PointHolme01-02 |  | 42.5° S, 147.9° E |  |  | SA, Vic & TAS | KT158855-6 | (Durrant et al., 2015) |
| *Ecklonia radiata* | Southern Tasmania, AU (4) | DennesPoint01-04 |  | 43.0° S, 147.3° E |  |  | SA, Vic & TAS | KT158804-7 | (Durrant et al., 2015) |
| *Ecklonia radiata* | Southern Tasmania, AU (5) | BlackmanBay01-05 |  | 43.0° S, 147.3° E |  |  | SA, Vic & TAS | KT158785-9 | (Durrant et al., 2015) |
| *Ecklonia radiata* | Southern Tasmania, AU (3) | GreenBluff01-03 |  | 42.7° S, 148.0° E |  |  | SA, Vic & TAS | KT158822-4 | (Durrant et al., 2015) |
| *Ecklonia radiata* | Southern Tasmania, AU (4) | HuonIsland01-04 |  | 43.2° S, 147.1° E |  |  | SA, Vic & TAS | KT158828-31 | (Durrant et al., 2015) |
| *Ecklonia radiata* | Southern Tasmania, AU (4) | LucasPoint01-04 |  | 43.3° S, 147.3° E |  |  | SA, Vic & TAS | KT158836-39 | (Durrant et al., 2015) |
| *Ecklonia radiata* | Western Tasmania, AU (3) | FarrellPoint03-05 |  | 43.3° S, 146.0° E |  |  | SA, Vic & TAS | KT158817-19 | (Durrant et al., 2015) |
| *Ecklonia radiata* | Western Tasmania, AU (3) | InnerSaddle01-03 |  | 43.0° S, 145.9° E |  |  | SA, Vic & TAS | KT158832-34 | (Durrant et al., 2015) |
| *Ecklonia radiata* | Western Tasmania, AU (1) | MuttonbirdIs3 |  | 43.4° S, 145.9° E |  |  | SA, Vic & TAS | KT158842.1 | (Durrant et al., 2015) |
| *Ecklonia radiata* | Central Vic, AU | KU-1134 |  |  |  |  | SA, Vic & TAS | AB775229.2 | (Kawai et al.2013) |
| *Ecklonia radiata* | South-east SA, AU (3) | SnapperNorth01-03 |  | 35.7° S, 138.0° E |  | 2015 | SA, Vic & TAS | KT158862-4 | (Durrant et al. 2015) |
| *Ecklonia radiata* | South-east SA, AU (5) | FlatIrons01-05 |  | 35.6° S, 138.5° E |  |  | SA, Vic & TAS | KT158812-6 | (Durrant et al., 2015) |
| *Ecklonia radiata* | South-east SA, AU (3) | GoatIsland01-03 |  | 37.1° S, 139.7° E |  |  | SA, Vic & TAS | KT158825-7 | (Durrant et al., 2015) |
| *Ecklonia radiata* | South-east SA, AU (5) | BaudinRocks01-05 |  | 37.0° S, 139.7° E |  |  | SA, Vic & TAS | KT158790-4 | (Durrant et al., 2015) |
| *Ecklonia radiata* | South-east SA, AU (5) | MypongaSth01-05 |  | 35.3° S, 138.3° E |  |  | SA, Vic & TAS | KT158843-7 | (Durrant et al., 2015) |
